# Intestinal Organoids—Current and Future Applications

**DOI:** 10.3390/vetsci3040031

**Published:** 2016-10-21

**Authors:** Andre M. C. Meneses, Kerstin Schneeberger, Hedwig S. Kruitwagen, Louis C. Penning, Frank G. van Steenbeek, Iwan A. Burgener, Bart Spee

**Affiliations:** 1 Institute of Animal Health and Production, Universidade Federal Rural da Amazônia, Avenida Presidente Tancredo Neves 66077-830, Brazil; 2 Department of Clinical Sciences of Companion Animals, Faculty of Veterinary Medicine, Utrecht University, Yalelaan 104, Utrecht 3584 CM, The Netherlands; k.schneeberger@uu.nl (K.S.); H.S.Kruitwagen@uu.nl (H.S.K.); L.C.Penning@uu.nl (L.C.P.); F.G.vanSteenbeek@uu.nl (F.G.v.S.); Iwan.Burgener@vetmeduni.ac.at (I.A.B.); B.Spee@uu.nl (B.S.)

**Keywords:** intestinal organoids, dog, practical applications

## Abstract

Recent technical advances in the stem cell field have enabled the in vitro generation of complex structures resembling whole organs termed organoids. Most of these approaches employ culture systems that allow stem cell-derived or tissue progenitor cells to self-organize into three-dimensional (3D)-structures. Since organoids can be grown from different species (human, mouse, cat, dog), organs (intestine, kidney, brain, liver), and from patient-derived induced pluripotent stem cells, they create significant prospects for modelling development and diseases, for toxicology and drug discovery studies, and in the field of regenerative medicine. Here, we report on intestinal stem cells, organoid culture, organoid disease modeling, transplantation, specifically covering the current and future uses of this exciting new insight model to the field of veterinary medicine.

## 1. Introduction

The intestinal tract is one of the most architecturally and functionally complex organs of the body [1] and the intestinal epithelium is an incredibly large mucosal surface with an extreme self-renewing capacity [2]. The canine intestine for medium sized dogs is about 4.5 m in length and is subdivided into five functional domains along the proximal-to-distal axis: the duodenum, jejunum, and ileum are segments of the small intestine, and the cecum and colon make up the large intestine [3].

In various pathological disorders or after medical treatments, such as chemotherapy and radiation therapy, the homeostasis between cell proliferation and cell death can become substantially disturbed, resulting in loss of epithelial integrity and local inflammation. The subsequent invasion of intestinal bacteria and the associated stimulation of the immune system further accelerate the course of the disease [4].

Recently a novel method that allows long-term culture of isolated intestinal crypts or intestinal stem cells was presented. Supplemented with the appropriate growth factor cocktail (epidermal growth factor, Noggin, R-spondin-1) and cultured in a three-dimensional extracellular matrix, these intestinal stem cells are capable of developing into organoids and enteroids, displaying many important functions of the normal intestinal epithelium (mini-guts) [5,6]. This culture model has been proven to serve as a powerful system to investigate regulatory and pathological mechanisms of the intestinal epithelium on a molecular level [4]. 

This organoid system has been extensively used for studying the regulation of intestinal stem cell self-renewal, growth, and differentiation. Additionally, in vitro expanded organoids may be used for gastrointestinal stem cell therapy in preclinical animal models, and in studies on colorectal tumor stem cells, among others [7].

## 2. Intestinal Stem Cells 

The small intestinal epithelium has enormous capacity for self-renewal, replacing itself every three to five days. The cellular basis for this regenerative potential has long been accepted to reside in multipotent intestinal stem cells (ISCs) [8]. ISCs reside near the bottom of the intestinal crypt. Their rapidly dividing, transit-amplifying (TA) daughter cells occupy the remainder of the crypts and flow onto the flanks of the villi, where they differentiate, absorb nutrients, and eventually die at the villus tips. The differentiated cell types include absorptive enterocytes, multiple secretory cells (Paneth cells, goblet cells, enteroendocrine cells, and tuft cells), and the M cells of Peyer’s patches [9].

Absorptive enterocytes are the predominant cell type in the intestinal epithelium and play a central role in the digestion and absorption of luminal nutrients. Goblet cells are the second most predominant cell type and produce mucin to lubricate the luminal surface. This mucin layer traps bacterial flora before they attach to the epithelial surface. Enteroendocrine cells are a rare cell type and are scattered along the cryptvillus axis. Upon sensing neuroendocrine signals, enteroendocrine cells produce various hormones to control digestive enzyme secretion, metabolism, and bowel movements. In contrast to these three differentiated cell types, Paneth cells are long-lived (two to three months) differentiated cells that migrate downward and dwell at the crypt bottom; there they sterilize the crypt lumen through the production of antibacterial peptides. The colonic epithelium is devoid of Paneth cells but harbors Paneth-like cells, named deep crypt secretory cells or crypt base goblet cells [10].

Two types of ISC were identified. The first, termed +4 cells, was defined on the basis of DNA label retention, proliferation, and radiation injury response, and the second, known as crypt base columnar (CBC) cells, was defined using cytological lineage tracing. More recently, CBC and +4 cells have been defined using molecular markers and transgenic lineage-tracing techniques. CBC cells are the proliferative engines that drive cellular production in the crypts: they divide daily, with frequent turnover. +4 cells have been redefined as reserve or quiescent ISCs (qISCs): they divide infrequently under homeostatic conditions but can be induced to produce new CBC cells in response to injury or other stimuli [11].

**Figure 1:**
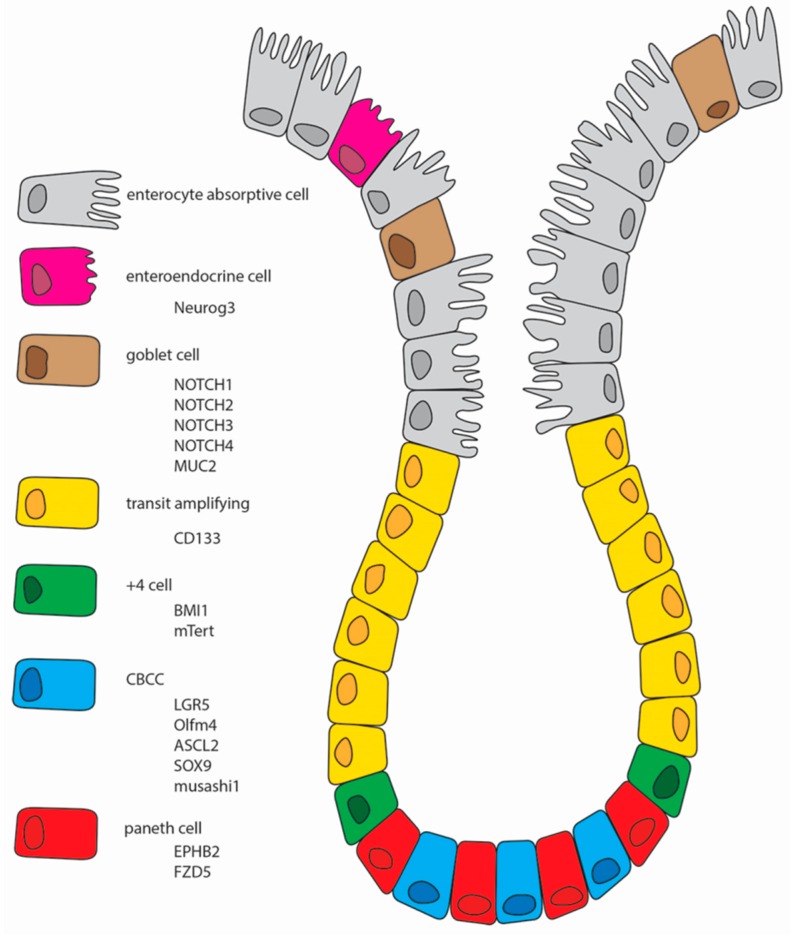
Schematic representation of the intestinal epithelium with all of the different cell types. **Neurog3**, Neurogenin3; **MUC2**, Mucin2; **CD133**, Prominin1; **BMI1**, B lymphoma Mo-MLV insertion region 1 homolog; mTert, Mouse telomerase reverse transcriptase; **CBCC**, crypt base columnar cells; **LGR5**, leucine-rich repeat-containing G-protein coupled receptor 5; **Olfm4**, Olfactomedin-4; **ASCL2**, homolog of the *Drosophila* Achaete-scute complex genes; **SOX9**, gene on chromosome 17q23 that encodes a member of the SOX (SRY-related HMG-box) family of transcription factors; **EPHB2**, gene on chromosome 1p36.1-p35 that encodes a member of the ephrin-B receptor subfamily of receptor tyrosine kinases; **FZD5**, Frizzled.

One of the reasons why the intestinal field has failed to reach a consensus regarding the identity and location of intestinal stem cells in the past four decades has been the lack of well-validated markers for this cell population. Such markers are essential for driving advances in our understanding of intestinal stem cell biology, tissue homeostasis, repair, and cancer [12]. There are several cells markers to the CBC stem cells and for the +4 cells [13]. Although their specificity is controversial, some of them will be presented below and are graphically summarized in Figure 1.

## 3. Markers of the Different Cell Types Present in the Intestinal Epithelium

Leucine-rich repeat-containing G-protein coupled receptor 5/G-protein coupled receptor 49 (Lgr5/GPR49) was originally identified as a Wingless-related integration site (Wnt) target gene encoding an orphan G-protein-coupled receptor in colorectal cancer cell-lines [13]. Investigation of its expression pattern in normal intestine revealed it to be a highly specific marker for the proliferating CBC cells [14]. The consideration that Lgr5 is a robust marker of intestinal stem cells was further substantiated by ex vivo culture assays, where single Lgr5^+^ cells were shown to form self-renewing epithelial organoids exhibiting a structure and composition highly reminiscent of crypt/villus epithelial units in vivo [6]. Importantly, cells that expressed low or no Lgr5 were incapable of forming these organoids. Taken together, these studies provide compelling evidence for Lgr5 being a marker of proliferating adult stem cells residing at the crypt base throughout the intestinal tract [13].

Bmi1 (B lymphoma Mo-MLV insertion region 1 homolog) is a member of the Polycomb group of transcriptional repressors, the function of which in the intestine is unknown. Bmi1 is an essential regulator of hematopoietic, neural, and lung epithelial stem cells. In the intestine, Bmi1 was initially detected in label-retaining stem cells located at the +4 position from the bottom of the crypt. This label retaining feature might indicate a quiescent nature or the capacity to asymmetrically segregate DNA strands. However, it was demonstrated that Bmi1 expression is unrestricted throughout the crypt compartment, similar to that of other proposed stem cell markers, such as Hopx (Homeodomain-only protein) and Tert (Telomerase reverse transcriptase). Furthermore, Bmi1 is a downstream effector of Notch in the ISC and progenitor compartment and Bmi1 is involved in ISC self-renewal [14].

CD133 (Prominin1 in mouse) was originally reported as a novel pentaspan glycoprotein expressed on neural and hematopoietic stem cells. More recently, it has been described as a marker of epithelial stem/progenitor populations in human kidney tubules and the prostate. However, a detailed analysis of reporter mice expressing lacZ under the control of the endogenous Prom1 promoter documented widespread expression in many adult epithelia, including the colon, casting doubt on its authenticity as a broad adult stem cell marker [13,15].

Telomerase is a ribonucleoprotein complex that helps maintain the telomeric ends of chromosomes, normally shortened with each cell division. Because loss of telomeric DNA beyond a critical threshold induces senescence in most somatic cells, maintenance or induction of telomerase activity provides a means of preventing cellular senescence that may be relevant for the self-renewal of tissue stem cells. Consistent with this hypothesis, loss of telomerase has been shown to result in intestinal villus atrophy, suggesting a functional requirement for telomerase activity and/or telomere maintenance in ISC function. mTert expression marks a slowly cycling ISC population distinct from Lgr5+ cells. mTert+ cells contribute to all differentiated intestinal cell types as well as the Lgr5+ cell population, persist long-term, are resistant to injury, and contribute to the regenerative response following tissue injury. Thus, a slowly cycling stem cell exists within the intestine alongside, and perhaps upstream of, the Lgr5+ population [16].

Olfactomedin-4 (Olfm4) has emerged from a gene signature for these Lgr5+ stem cells as a robust marker for murine small intestinal stem cells. Studies have shown that OLFM4 is highly expressed in crypt base columnar cells in human small intestine and colon. Moreover, it is expressed in cells within adenocarcinomas of the colon and can serve as a useful marker for Lgr5-type stem cells in human small intestine and colon [17]. Oflm4 is a secreted molecule that is specifically and robustly expressed on the stem cells of the small intestine, but is absent from the colon in mice. In contrast to Lgr5, expression of Oflm4 is not under the control of the Wnt pathway [13].

Ascl2 is a homolog of the *Drosophila* Achaete-scute complex genes and is a Wnt target gene. The Ascl2 gene encodes a basic helix-loop-helix (bHLH) transcription factor with an unusually restricted expression pattern (i.e., its expression is predominantly detected in extraembryonic tissues and in intestinal epithelium) [18]. Ascl2 has been proposed to be a master regulatory gene for Lgr5+ ISCs, since transgenic overexpression in mice induces hyperplastic and ectopic crypts in the villus compartment, while its deletion results in loss of Lgr5+ ISCs [19].

SOX transcription factors have the capacity to modulate stem/progenitor cell proliferation and differentiation in a dose-dependent manner. SOX9 is expressed in the small intestine epithelial stem cell zone and has been reported to be a downstream target of Wnt signaling by acting in a feedback loop to repress Wnt signaling, thus keeping proliferation under tight regulatory control. Distinct levels of SOX9 in the intestinal epithelium are associated with both proliferative and postmitotic cell types and, furthermore, these variable SOX9 levels likely play an important role in both the control of proliferative capacity of stem/progenitor populations and also the maturation of enteroendocrine cells, where low SOX9 expression supports proliferative capacity, and high SOX9 expression suppresses proliferation [20]. 

The receptor tyrosine kinase EphB2, is expressed in a decreasing gradient from the crypt base toward the differentiated cell compartment. EphB activity is required to establish the position of the different cell types in the crypts and the EphB2 expression gradient is required to compartmentalize cell populations in different territories of the healthy epithelium [21].

Wnt/b-catenin signaling is crucial for normal stem cell function in the intestinal epithelium. More specifically, Wnt3 signaling, provided by flanking Paneth cells, is necessary for the maintenance and function of CBC stem cells. In the absence of Wnt3, Wnt2b can compensate. The weak short range Wnt signal is augmented by R-spondin signaling through Lgr receptors. R-spondins are incorporated into a complex that contains Lrp (low-density lipoprotein receptor related proteins), Lgr, and Fzd (Frizzled); this complex facilitates Fzd-coupled Wnt/b-catenin signaling [22]. Of the ten mammalian Fzds, only Fzd7 is frequently upregulated in stem cell populations and cancers from diverse tissues [23]. Notably, FZD7 plays a non-redundant role in maintaining pluripotency of human embryonic stem cells [24] and might play a similar role in the intestinal stem cells. It was demonstrated that intestinal stem cells that do not express Fzd7 have an inherent defect in Wnt/b-catenin signaling, which compromises stem cell function under conditions of stress [22].

Neurogenin 3 (NEUROG3) has been investigated as a candidate gene responsible for congenital loss of intestinal enteroendocrine cells in humans because of its known role in enteroendocrine cell development in mouse [25]. The intestinal enteroendocrine cells consist of at least 15 different cell types classified essentially on the basis of their hormonal content with a specific geographical distribution. NEUROG3 is expressed in the fetal intestinal epithelium as well as immature cells located at the proliferative compartment of the crypts in the adult small intestine [26].

Notch is clearly active in ISCs and stimulates proliferation while blocking secretory cell differentiation. Furthermore, Notch is a direct activator of the ISC gene Olfm4 and the bHLH transcriptional repressor Hes1, which in turn blocks expression of the driver of secretory cell differentiation, Math1. The co-operation between Notch and Wnt in the intestine results in the amplification of Wnt-driven proliferation and Wnt-driven tumor formation is potentiated by constitutive Notch activation [27]. Notch is likely to target distinct stem and progenitor cell populations to regulate different aspects of intestinal homeostasis, although specific cellular targets have not been definitively identified. Crucial components of the Notch signaling pathway, including the Notch1 and Notch2 receptors, the ligands jagged 1, Dll1 and Dll4, and the Notch target genes hairy and enhancer of split 1 (Hes1), Hes5 and Hes6, have been localized to the proliferative zone of the intestinal crypts. Importantly, lineage tracing from cells undergoing active Notch signaling identified long-lived progenitors that gave rise to all the mature epithelial cell types, suggesting that Notch signaling was active in a stem cell. More specifically, Notch regulation of the CBC stem cell was suggested by the enrichment of Notch1 receptor mRNA in this cell type [28].

MUC2 encodes a large (>5000 amino acid residue) gel-forming mucin abundantly expressed by intestinal goblet cells and certain other mucus-secreting cells in normal, diseased, and neoplastic tissues. The 58-flanking region of the MUC2 gene has been isolated as well, and has been shown to have promoter activity in cultured cells. It is difficult to study the factors responsible for the regulation of this gene in cultured cells, however, in part because transfectable cell lines expressing the high levels of MUC2 found in intestinal goblet cells are nonexistent [29].

The RNA-binding protein Musashi 1 (Msi1), a regulator of asymmetric cell division, is also involved in stem cell maintenance. Particularly, in neural stem cells Msi1 is able to maintain stemness properties through Notch pathway activation. Independent immunoistochemical and in situ hybridization analyses demonstrated that Msi1 is expressed in the CBC cells immediately above the Paneth cells. Moreover, Msi1 overexpression in the intestine increases both Wnt and Notch pathways, and induces the upregulation of Lgr5 and Bmi1. Interestingly, although Msi1 is expressed in putative ISCS, in knockout mice lacking this marker, no defects in the development of the intestine are detected. Taken together, these observations demonstrated that Msi1 is not a specific ISCS marker, but is expressed in both ISCS and in their early progeny [7].

## 4. Organoid Culture

Currently, the two primary ways to generate human mini intestines include (a) isolation of intestinal crypts, which contain human adult stem cells, from donors or (b) use of human embryonic or inducible pluripotent stem cells (iPSCs). Intestinal crypts, containing human adult stem cells, can be isolated either from surgically resected tissue or from biopsies, embedded in Matrigel, and cultured as ex vivo self-perpetuating three-dimensional primary cultures in growth factor enriched media [30]. 

Initially, these cultures produce polarized three-dimensional spheroid-like structures with the apical domain facing inside newly formed lumens and basolateral surfaces in contact with the Matrigel and external media. These are referred to as enteroids (derived from small intestine) or colonoids (derived from colon) and contain only epithelial cells types derived from the crypt-based stem cells. Human three-dimensional intestinal tissue cultures, termed organoids, can also be generated in vitro from iPSCs [30]. Intestinal organoids are generated from iPSCs that are differentiated to form mid- and hindgut tissue. From the mid- or hindgut epithelial cell layers, three-dimensional spheroid buds are further cultured in Matrigel along with pro-intestinal growth factors [25].

Organoids have been successfully generated from many regions of the mouse gastrointestinal tract, ranging from the tongue through to the colon Recently, human iPSC-derived functional cholangiocyte organoids have also been generated by the modulation of the Activin A and Notch signaling pathways. A notable difference between organoids derived from primary tissue and ESCs/iPSCs is the presence of cell types other than the intended lineage in the latter. This is because the factors used for directed differentiation of ESCs/iPSCs are not completely efficient in driving all the cells towards the lineage of choice, thus many ectodermal and endodermal organoids, such as those of the intestine, stomach, and kidney, have reported the limited presence of mesenchymal cell type [31].

Although a variety of culture systems have been described, only recently have long-term culture systems become available that maintain basic crypt physiology, based on the growth factor requirements observed in vivo. A Matrigel based culture system was established that allows the formation of ever-expanding organoids, or ‘‘mini-guts’’, in vitro from a single Lgr5+ stem cell [6]. 

An essential component of these cultures is the Wnt agonist Rspondin1, the ligand of Lgr5. The other constituents are epidermal growth factor (EGF) and the bone morphogenetic protein (BMP) inhibitor Noggin [2]. For colon crypt culture, Wnt ligand is an additional factor required to maintain Lgr5-CBC cells, because the epithelium makes little, if any, Wnt [9]. It has been suggested that some of these growth factors in small intestine are derived from the neighboring Paneth cells in vivo, while other cues are likely provided by intestinal subepithelial myofibroblasts (ISEMFs). Paneth cells are tightly nested between ISCs in the crypt base; they act as anti-microbial defensive cells, while also producing supportive growth factors for neighboring ISCs. It has been reported that Paneth cell-derived Wnt3 is necessary for in vitro intestinal epithelial cultures. However, ISC niche maintenance in vivo is preserved even with conditional epithelial Wnt3 deletion and Paneth cell ablation. This suggests the presence of redundant sources of Wnt signaling from surrounding non-epithelial cells within the niche [32].

The mini-guts faithfully recapitulate the central features of normal small intestinal epithelium. They consist of crypts (with resident Lgr5 cells, Paneth cells, and TA cells) that feed into a central lumen lined by mature epithelial cells of all villus lineages. Self-renewal kinetics resemble the in vivo situation: cells are born in the crypts proliferate, differentiate, and are shed into the central lumen about five days later [2]. The isolated crypts require Matrigel (BD Biosciences, San Jose, CA, USA), a 3D laminin-and collagen-rich matrix that mimics the basal lamina. Single crypts can be readily isolated from mouse or human intestine by ethylenediaminetetraacetic acid (EDTA)-based Ca2+/Mg2+ chelation [9], as well as in dogs (data not shown).

In vitro-generated organoids occur as cysts with a central lumen flanked by a simple, highly polarized villus epithelium. Multiple cryptlike structures project outward. The basal side of the cells is oriented toward the outside, touching the matrigel, whereas enterocyte brush borders form the luminal surface. Secretion by Paneth and goblet cells occurs toward the lumen. The organoids can be passaged weekly at a 1:5 ratio for at least 1.5 years, with a phenotype and karyotype that remain unchanged. Mechanically disrupted organoids rapidly reseal. Self-renewal kinetics and cell-type composition closely resemble the in vivo situation in small intestinal epithelial derived organoids. Notably, a timer of unknown molecular nature remains active in the absence of the in vivo wear-and-tear: two to three days after terminal differentiation, the cells exfoliate into the lumen [9].

In a medium containing Wnt3A in addition to R-spondin-1 (Rspo1), the sharp Wnt gradient is lost and epithelial mini-guts become symmetric, round cysts, consisting of a homogeneous population of stem and progenitor cells. Epithelial mini-guts grown from adenomatous polyposis coli (APC)—mutant adenoma cells display the same symmetric shape, which is not surprising, because APC loss leads to constitutive Wnt pathway activation. These observations imply that the typical crypt-villus architecture is suppressed under conditions of homogeneous (rather than focal) Wnt signaling [33]. The recently published immobilized Wnt protein can be used to improve intestinal stem cell niche architecture [34]. 

The addition of Rspo1, Noggin, and epidermal growth factor (EGF), which are all essential to small intestine culture, did not maintain the growth of mouse colonic crypts. Therefore authors developed the following “TMDU (Tokyo Medical and Dental University) protocol”: crypts were embedded in type I collagen in serum-free medium with Wnt3a, hepatocyte growth factor (HGF), and BSA, in addition to Rspo1, Noggin, and EGF. Although Noggin, EGF, and HGF were not essential for growth of the colonic crypts, each enhanced their growth [35].

When a single cultured stem cell is followed over time, a small symmetric cyst forms. The stochastic appearance of a Paneth cell constitutes the “symmetry-breaking” event: a bud forms around the cell; within two to three days this bud develops into a cryptlike structure with stem and Paneth cells. Every nondifferentiated cell that touches a Paneth cell is (or becomes) an Lgr5- CBC cell, presumably driven by potent Wnt and Notch signals from the Paneth cell [8,33]. The proliferative stem cell—Paneth niche pushes itself outward from the central cyst. This process is most likely driven by repulsive EphB-EphrinB interactions, as described in vivo [35]. 

Although mouse models and genetic analyses have greatly increased the general understanding of embryonic gastrointestinal (GI) development, mouse development does not always recapitulate human [33] development, particularly in development of the intestinal stem cell niche.

Organoids have been described in numerous tissues form humans and mice [36]; regarding companion animals, only one paper described canine liver organoids [37]. Since growth factor dependency for the various organ/species derived organoids varies greatly, the time needed to discover the optimal culture conditions can be cumbersome. 

## 5. Organoid Disease Modeling and Transplantation

Gastrointestinal (GI) organoids have been used to model human genetic diseases, infections, inflammatory bowel diseases (IBD), and malignancies. They can be used too to conduct drug screens and identify personalized approaches for treating disease [38].

Also of interest is the direct use of intestinal organoids as therapy. Intestinal organoids have been transplanted into damaged colon for tissue repair [35], but only with limited engraftment success [39].

Enteroids are showing great promise as models to study the interaction between intestinal pathogens and the intestinal epithelium. To date, human enteroids have been shown to model human rotavirus (RV) infection, cholera toxin effect on transport, and several aspects of enterohemorrhagic *Escherichia coli*-related human diarrhea. RV replicates and produces infectious viruses in human enteroids, with viral replication increasing over 96 h [30].

The evolution of the enteroid/organoid models to include sub-epithelial myofibroblasts, smooth muscle cells, enteric nerves, immune cells, microbiome, and endothelial cells is expected to help facilitate our understanding of complex intestinal organ development. In addition, methods have been established to alter gene expression either by overexpression or shRNA-mediated knockdown using lipophilic transfection reagents and virus mediated transduction [40,41].

These new models may provide valuable insight into developing methodologies for intestinal regeneration that lead to therapeutic options including repair of the damaged epithelium that occurs in ulcerative colitis, microvillus inclusion disease, or intestinal transplantation to treat short bowel syndrome [42]. Despite these evolutions in therapeutic possibilities, the in vivo transplantation in humans remains a challenge [43].

In spite of the successful rodent studies [44], successful generation of neomucosa using an intestinal cell-cluster in transplantation was first described in dogs in 2009. The authors showed that the isolation and transplantation of 3D cultured cells can be used to generate neomucosa in a dog model [45]. However the molecular characterization of the presumed “organoids” was not rigorously described. 

The intestinal organoids can be utilized not only to investigate the crucial aspects of gastrointestinal biology, but their usage could also facilitate functional studies, diagnoses, and personalized treatments of various disorders including cancer [46]. Deregulation of Wnt signaling is associated with onset of cancer, most notably carcinoma of the colon and rectum [46]. Other studies have employed CRISPR/Cas9-mediated gene editing of healthy organoids to directly evaluate candidate gene function in tissue physiology and carcinogenesis [47].

Despite the strengths that organoids offer over traditional cell lines, their use is restricted. For example, although organoids are complex and often retain multiple cell lineages, they lack many of the cellular inputs present in an in vivo system. Complexity can be increased through coculture with additional cell types, but organoids should still be viewed as a reductionist system [35].

Recently the first generation of 3D human gastric organoids was reported through directed in vitro differentiation of hESCs and hiPSCs. The authors recapitulated in vivo human stomach development by inducing first the PSCs to form definitive endoderm spheroids, which were subsequently stimulated by retinoic acid to generate the foregut. To permit 3D growth and further differentiation, spheroids were transferred into a retinoic acid-supplemented Matrigel culture, leading to the formation of domains with gastric mucous and endocrine cells, and LGR5-expressing SCs. Notably, to study the etiology of *Helicobacter pylori*-mediated disease, they injected the bacterium directly into the lumen of the organoids, and observed that the virulence factor CagA bound and activated the c-Met (receptor for HGF), inducing epithelial proliferation. This epithelial pathophysiological response makes the organoids a promising model for human gastric disease [48].

Transplanted human intestinal organoids have displayed mature intestine characteristics such as peptide uptake, but therapeutic application would require additional functions, such as the ability to perform the peristaltic movements essential for food bolus transition along the digestive tract [49].

## 6. Future of Organoids in Veterinary Medicine 

Despite the great advances in the field of intestinal organoids, the great challenge is, as described by Fatehullah et al. [48], to rapidly and safely translate the knowledge of mouse stem cells into the human arena. Efforts to exploit intestinal stem cells for regenerative medicine have accelerated over the past decade, driven mostly by the identification of cell-surface markers facilitating the isolation of pure stem cell populations and the development of near-physiological culture methods supporting stem cell expansion and epithelial growth. To keep momentum and to ensure safety issues in the mouse-to-human translation, dogs are ideally suited as experimental animals and clinical patients, having GI- and liver-pathologies highly similar to humans.

With their robust genomic integrity, it will be feasible to use organoids as a tool for regenerative medicine and biomedical research, and in the near future create tissues for autologous transplantation. Besides that, the development of “cancer organoids” creates significant prospects for personalizing and optimizing current treatments [49].

Studies involving molecularly characterized intestinal organoids in dogs are lacking. Because of that, a task-force at the Faculty of Veterinary Medicine of the Utrecht University (Utrecht, the Netherlands) was created to fill this gap. Recent studies have shown that maintenance of canine intestinal organoids are workable (data not published) and that practical applications can be tested, as for the development and study of viral infections such canine parvovirus and coronavirus. This system can be also used for other species like porcines, equines, ruminants, and felines, but studies in these species are punctual and scarce, and advances and technique adaptations will be necessary to use this method for them.

In the future, organoid technologies will undoubtedly provide a methodological window, allowing us to understand development in all species, including companion animals, and disease in depth, and will enabling new therapeutic options and a better understanding of the pathophysiological processes of various diseases.

## Abbreviations

The following abbreviations are used in this manuscript:

ISCs: Intestinal stem cells
TA: transit-amplifying cells
CBC: crypt base columnar cells
qISCs: quiescent intestinal stem cells
LGR5: Leucine-rich repeat-containing G-protein coupled receptor 5
Bmi1: Proto-Oncogene, Polycomb Ring Finger
Hopx: Homeodomain-only protein
Tert: Telomerase reverse transcriptase
Prom1: Prominin 1
Olfm4: Olfactomedin-4
Ascl2: homolog of the *Drosophila* Achaete-scute complex genes
bHLH: basic helix-loop-helix
SOX9: A gene on chromosome 17q23 that encodes a member of the SOX (SRY-related HMG-box) family of transcription factors
EphB2: gene on chromosome 1p36.1-p35 that encodes a member of the ephrin-B receptor subfamily of receptor tyrosine kinases
Lrp: low-density lipoprotein receptorrelated proteins
Fzd: Frizzled
Neurog3: Neurogenin3
bHLH: basic helix-loop-helix
Hes1: hairy and enhancer of split-1
Math1: neural-specific basic helix-loop-helix transcription factor
mRNA: Messenger RNA
MUC2: Mucin2
Msi1: Musashi 1
Rspo1: R-spondin-1
EGF: Epidermal growth factor
BMP: Bone Morphogenetic Proteins
ISEMFs: Intestinal subepithelial myofibroblasts
EDTA: Ethylenediaminetetraacetic acid
APC: adenomatous polyposis coli
TMDU: Tokyo Medical and Dental University
HGF: Hepatocyte growth factor
GI: Gastrointestinal
IBD: Inflammatory bowel diseases
RV: Rotavirus
hESC: human embryonic stem cell
hiPSCs: human-induced pluripotent stem cells
PSCs: pluripotent stem cells
CagA: cytotoxin-associated gene A
c-MET: MET proto-oncogene, receptor tyrosine kinase
